# ATTED-II in 2016: A Plant Coexpression Database Towards Lineage-Specific Coexpression

**DOI:** 10.1093/pcp/pcv165

**Published:** 2015-11-06

**Authors:** Yuichi Aoki, Yasunobu Okamura, Shu Tadaka, Kengo Kinoshita, Takeshi Obayashi

**Affiliations:** ^1^Graduate School of Information Sciences, Tohoku University, 6-3-09, Aramaki-Aza-Aoba, Aoba-ku, Sendai, 980-8679 Japan; ^2^Core Research for Evolutional Science and Technology (CREST), Japan Science and Technology Agency, Kawaguchi, Saitama, Japan; ^3^Institute of Development, Aging, and Cancer, Tohoku University, Sendai, 980-8575 Japan; ^4^Tohoku Medical Megabank Organization, Tohoku University, Sendai, 980-8573 Japan

**Keywords:** Arabidopsis, Comparative transcriptomics, Database, Evolution, Gene coexpression, Gene network

## Abstract

ATTED-II (http://atted.jp) is a coexpression database for plant species with parallel views of multiple coexpression data sets and network analysis tools. The user can efficiently find functional gene relationships and design experiments to identify gene functions by reverse genetics and general molecular biology techniques. Here, we report updates to ATTED-II (version 8.0), including new and updated coexpression data and analysis tools. ATTED-II now includes eight microarray- and six RNA sequencing-based coexpression data sets for seven dicot species (Arabidopsis, field mustard, soybean, barrel medick, poplar, tomato and grape) and two monocot species (rice and maize). Stand-alone coexpression analyses tend to have low reliability. Therefore, examining evolutionarily conserved coexpression is a more effective approach from the viewpoints of reliability and evolutionary importance. In contrast, the reliability of species-specific coexpression data remains poor. Our assessment scores for individual coexpression data sets indicated that the quality of the new coexpression data sets in ATTED-II is higher than for any previous coexpression data set. In addition, five species (Arabidopsis, soybean, tomato, rice and maize) in ATTED-II are now supported by both microarray- and RNA sequencing-based coexpression data, which has increased the reliability. Consequently, ATTED-II can now provide lineage-specific coexpression information. As an example of the use of ATTED-II to explore lineage-specific coexpression, we demonstrate monocot- and dicot-specific coexpression of cell wall genes. With the expanded coexpression data for multilevel evaluation, ATTED-II provides new opportunities to investigate lineage-specific evolution in plants.

## Introduction

Identifying similarities in the expression profiles of different genes, or coexpression, can provide insight to elucidate gene function (Eisen et al. 1998, Walker et al. 1999). Backed up by the enlargement of public gene expression repositories, the usefulness of coexpression information has been expanding (Rung and Brazma 2013). Because the biological functions of paralogous genes are not clearly distinguished by their sequence similarities, a gene coexpression database is a prominent resource to estimate gene function, especially in plants, which generally have more paralogous genes than animals (Tang et al. 2008). The quality of coexpression data is primarily based on the number of samples (Ballouz et al. 2015), and this characteristic limits application of coexpression analysis in non-model species. However, recent technical advancements in RNA sequencing (RNAseq) are overcoming this difficulty. During the last decade, gene coexpression databases have been constructed and used for a wide variety of species (Aoki et al. 2007, Usadel et al. 2009).

With the maturation of coexpression data, meta-analyses of coexpression are becoming increasingly important. Coexpression analysis based on a single platform will have technical biases associated with that platform. For example, the properties of cross-hybridization and the dynamic range of probes differ among microarray platforms, as does the signal-to-noise ratio. These technical biases can lead to false positives in the coexpression analysis. Therefore, multiple platform comparisons for a single species are an effective way to eliminate false positives. Examination of coexpression conservation between closely related species has similar benefits. In addition, comparisons between evolutionarily distant species have highlighted the evolutionary conservation of coexpression, which supports the functional relationship of the gene pairs, rather than the technical reliability (Stuart et al. 2003, Oti et al. 2008, Movahedi et al. 2011, Okamura et al. 2015). Some coexpression databases allow assessment of coexpression conservation (Obayashi and Kinoshita 2011, Obayashi et al. 2011).

Species-specific co-expression has also attracted researchers with an evolutionary viewpoint (Stuart et al. 2003, Oldham et al. 2006), but false species-specific relationships can also be generated by the technical bias of the platform or by different sample compositions. Therefore, species-specific coexpression analysis always requires evidence that the results are independent from both the platform characteristics and the sample composition.

We constructed and developed ATTED-II (http://atted.jp), a coexpression database for plant species, which provides a parallel view of multiple coexpression data sets with network analysis tools (Obayashi et al. 2007, Obayashi et al. 2009, Obayashi et al. 2011, Obayashi et al. 2014). The user can effectively find functional gene relationships and design experiments to confirm the gene functions by reverse genetics and general molecular biological techniques (Obayashi and Kinoshita 2010). Here, we report updates to ATTED-II that include new and updated coexpression data and analysis tools. ATTED-II now includes eight microarray- and six RNAseq-based coexpression data sets for nine species (Arabidopsis, field mustard, soybean, barrel medick, poplar, tomato, grape, rice and maize). Importantly, five species (Arabidopsis, soybean, tomato, rice and maize) are now supported by both microarray- and RNAseq-based coexpression data. Our assessment scores for the data indicate that the new coexpression data sets are of higher quality than any previous coexpression data sets in ATTED-II. These highly reliable coexpression data will enable us to detect lineage-specific coexpression. As an example, we demonstrate monocot- and dicot-specific coexpression with the updated ATTED-II. With the expanded coexpression data with multilevel evaluation, ATTED-II provides new opportunities to investigate lineage-specific evolution in plants.

## Results and Discussion

### The new co-expression data for nine species from 14 sources

We updated both the microarray- and RNAseq-based coexpression data (Table 1) in ATTED-II. For the microarray platform, tomato (*Solanum lycopersicum* microarray; Sly-m) was newly included, and additional microarray data for Arabidopsis (*Arabidopsis thaliana*; Ath-m), soybean (*Glycine max*; Gma-m), barrel medick (*Medicago truncatula*; Mtr-m), rice (*Oryza sativa*; Osa-m) and grape (*Vitis vinifera*; Vvi-m) were downloaded from a public repository (Kolesnikov et al. 2015). For RNAseq-based coexpression data, field mustard (*Brassica rapa* RNAseq; Bra-r), soybean (Gma-r), rice (Osa-r), tomato (Sly-r) and maize (*Zea mays*; Zma-r) were newly added, and the Arabidopsis coexpression data were updated (Ath-r). In total, ATTED-II provides information from 14 sources for the nine species. Among the nine species, five (Arabidopsis, soybean, tomato, rice and maize) are supported by data from both the microarray and RNAseq platforms, which enhances the reliability of the coexpression detection in these species described below.

**Table 1:** Coexpression data in ATTED-II version 8.0

Common name	Scientific name	Data set ID*^a^*	Version	No. of genes	No. of samples	Codon score*^b^*	Reproducibility score*^c^*	Release date
Arabidopsis	*Arabidopsis thaliana*	Ath-m	c6.0	20,836	15,275	2.29	2.57	August 31, 2015
		Ath-r	c2.0	25,296	1,401	1.67	2.20	August 31, 2015
Field mustard	*Brassica rapa*	Bra-r	c1.0	35,431	257	1.63	1.25	August 31, 2015
Soybean	*Glycine max*	Gma-m	c2.0	15,902	1,115	1.63	1.61	August 31, 2015
		Gma-r	c1.0	42,787	410	1.73	1.48	August 31, 2015
Barrel medick	*Medicago truncatula*	Mtr-m	c2.0	6,226	909	1.08	–	August 31, 2015
Rice	*Oryza sativa*	Osa-m	c5.0	20,625	2,098	1.59	1.79	August 31, 2015
		Osa-r	c1.0	17,548	222	1.49	1.64	August 31, 2015
Poplar	*Populus trichocarpa*	Ppo-m	c1.0	21,909	404	1.29	1.59	May 23, 2013
Tomato	*Solanum lycopersicum*	Sly-m	c1.0	5,786	401	1.07	1.51	August 31, 2015
		Sly-r	c1.0	23,195	288	1.34	1.27	August 31, 2015
Grape	*Vitis vinifera*	Vvi-m	c2.0	9,564	245	1.18	1.32	August 31, 2015
Maize	*Zea mays*	Zma-m	c2.0	11,069	755	1.62	1.94	August 31, 2015
		Zma-r	c1.0	22,592	1,571	2.55	1.85	August 31, 2015

*^a^* -m, microarray-based coexpression data; -r, RNAseq-based coexpression data.

*^b^*Coincidence score between coexpressed gene lists and codon similarity gene lists. The agreement between the two types of gene lists is quantified using the COXSIM value for each guide gene. The median COXSIM value (1E-02) was used to assess overall performance. A higher score indicates better performance.

*^c^*Coincidence score with reference data sets represented by the median of the normalized COXSIM value (1E-01). A higher score indicates better performance.

### Similarity among the 14 coexpression data sets

**Fig. 1:**
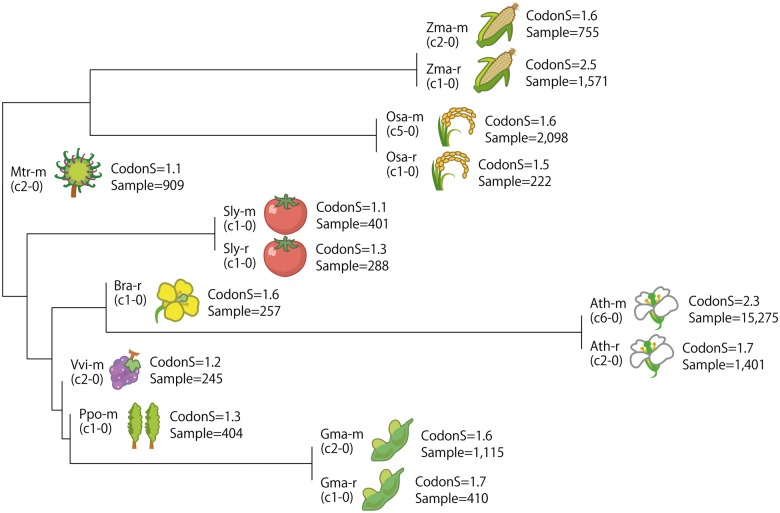
Hierarchical clustering of coexpression data. Data sets were hierarchically clustered by the complete linkage method. The pairwise similarities among all coexpression data sets are shown in Supplementary Fig. S1. Because COXSIM values are not exactly symmetric, the median of the COXSIM for a pair is not exactly symmetric. Therefore, the average values of the median COXSIM between one target and one reference, and vice versa, were used to represent symmetric similarity between data sets, and 1 – similarity was used to represent the distance between data sets. The coexpression data set version is shown in parentheses under the data set ID. ‘CodonS’ is the codon score from Table 1. ‘Sample’ indicates the number of samples in the data set.

To provide an overview of the 14 coexpression data sets, we examined the similarities among them. We first quantified the similarity between pairs of coexpressed gene lists from different sources using the coexpression similarity (COXSIM) value, which is the weighted concordance rate of a gene list (Obayashi et al. 2013). Because the COXSIM values are calculated for every pair of corresponding guide genes, the median of the COXSIM values for all guide gene pairs was used to represent the similarity of the two coexpression data sets. Supplementary Fig. S1 shows the similarities among the 14 coexpression data sets. Among all data set pairs, the five pairs from the same species (Ath, Gma, Sly, Osa and Zma) showed the highest similarities, as expected. In contrast, the Mtr-m coexpression data showed similarity only to the Gma-r data (0.018), probably owing to the low quality of the barrel medick data. The similarity table is also represented as a dendrogram in Fig. 1. In the dendrogram, the high similarities of the platform pairs (microarray and RNAseq) for the five species are represented as the longest branches, whereas barrel medick, which did not show strong similarity to the other platforms, was placed near the root of the dendrogram. Importantly, this dendrogram reflects the phylogenetic branching of the brassica family (Ath and Bra) and the monocot–dicot branching, suggesting the potential to analyze the evolution of coexpression.

### Significance of the coexpressed gene list

**Fig. 2:**
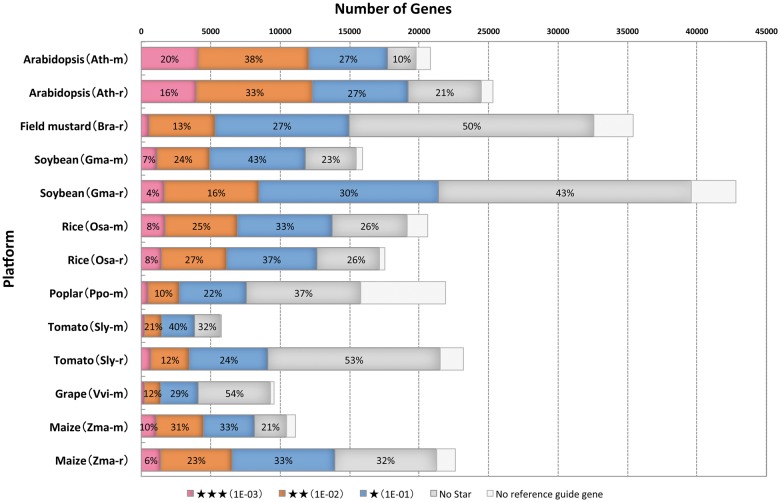
Number of guide genes for each supportability level. Supportability levels are represented as stars, where no star is the lowest and three stars is the highest. The numbers in the color-coded bars indicate the percentage of genes in each supportability level in each data set. Genes without any reference genes in the other data sets are shown as blank boxes.

When coexpression is supported by data from multiple platforms in the same species or closely related species, the coexpression can be regarded as reliable. Because selection of the best platform to assess the coexpressed gene list of interest depends on multiple factors, we used the maximum COXSIM value (maxCOXSIM) between the target gene list and each reference gene list as the measure of supportability of the target coexpressed gene list (Obayashi et al. 2013). The maxCOXSIM was then compared with the null distribution to calculate the statistical significance. We slightly refined the null distribution of the maxCOXSIM to estimate a more realistic *P*-value. The previous null distribution of maxCOXSIM was constructed by randomization of the individual gene list. However, the actual coexpressed gene list has two types of constraints that reduce the degrees of freedom. One constraint concerns the characteristic of correlation. When both of the two variable pairs A–B and B–C are correlated, the pair A–C is not independent. The other constraint concerns gene expression patterns. Compared with the conceptually possible variation in gene expression patterns, actual gene expression patterns in cellular systems are very limited. Therefore, independent randomization of the gene list allows too many degrees of freedom and consequently leads to overestimated *p*-values. To achieve more realistic *p*-values of maxCOXSIM, we used the distribution of the actual COXSIM values between any combination of guide genes of the Arabidopsis (Ath-r) and rice (Osa-r) data sets as the null distribution of the COXSIM values (Supplementary Fig. S2). Based on this COXSIM distribution, the thresholds of maxCOXSIM were defined, after Bonferroni correction, for 13 guide gene lists. The thresholds of maxCOXSIM for *p* < 0.1, *p* < 0.01 and *p* < 0.001 are 0.081, 0.189 and 0.377, respectively, and are represented as one, two and three stars on the ATTED-II coexpressed gene page. As an example, the supportabilities for the coexpressed gene list of CS6/At5g64740 are available in http://atted.jp/cgi-bin/coex_list.cgi?gene=836595. The proportions of genes of each significance level in each data set are shown in Fig. 2. This supportability analysis suggests that Arabidopsis, soybean, rice and maize are the best species for coexpression analysis.

### Performance of overall gene coexpression data

We then assessed the 14 sets of coexpression data using three independent scores: (i) the Gene Ontology (GO) score; (ii) codon score; and (iii) reproducibility score. Because rich gene annotation resources are available for Arabidopsis, we first tested for enrichment of the GO biological process annotations (GO score; Obayashi et al. 2014). The GO scores showed improvement with each update of the Arabidopsis coexpression data (Table 2). Compared with the improvement seen with addition of RNAseq data (Ath-r), the improvement with the addition of microarray data (Ath-m) from c5-0 to c6-0 was small, implying saturation of sample variation for this platform. Although the GO score is informative for Arabidopsis, this score is not generally applicable for the numerous species that lack comprehensive GO annotations (Obayashi et al. 2014).

**Table 2:** Consistency of the three assessments for Arabidopsis coexpression data in ATTED-II

Data set ID	Version	No. of genes	No. of samples	GO score*^a^*	Codon score*^b^*	Reproducibility score*^c^*	Release date
Ath-m	c6.0	20,836	15,275	7.08	2.29	2.57	August 30, 2015
	c5.0	20,836	11,171	7.02	2.24	2.55	May 17, 2013
	c4.1	20,906	1,388	5.48	1.56	2.08	April 8, 2008
	c4.0	20,906	1,388	5.06	1.59	1.82	March 18, 2008
	c3.1	20,703	771	4.96	1.46	2.14	September 12, 2007
	c3.0	22,263	771	4.96	1.46	2.10	May 25, 2006
Ath-r	c2.0	25,296	1,401	4.81	1.67	2.20	August 30, 2015
	c1.0	25,838	328	4.27	1.59	1.67	August 17, 2013

*^a^*Predictive performance of the GO annotation represented by AUC_0.01_ (1E-04). A higher score indicates better performance.

*^b^*Coincidence score with codon similarity represented by the median of the COXSIM value (1E-02). A higher score indicates better performance.

*^c^*Coincidence score with reference data sets represented by the median of the normalized COXSIM value (1E-01). A higher score indicates better performance.

As the second assessment, we determined the codon score, which is a coincident score between a coexpressed gene table and a gene–gene codon usage similarity table (Obayashi et al. 2014). Because codon usage information is available for genes in any species, the codon score can be applied to any coexpression data. The codon scores (Table 2) also showed general improvement with addition of Arabidopsis coexpression data, in good agreement with the GO scores [Pearson’s correlation coefficient (PCC) = 0.91].

The third assessment score is based on the similarity of the coexpression data sets (Fig. 1; Supplementary Fig. S1). As discussed above, reproducible coexpression data can be assumed to be of high quality. However, to use supportability directly as a measure of overall quality of a data set, the quality of the reference data set should be considered. Generally, lower quality reference data do not affect maxCOXSIM, which is the highest COXSIM value among all reference data sets. In most cases, taking the maximum value means selecting the data sets of the closest or the same species if available. However, the data set of the closest species is not always the best choice in terms of similarity assessment. For example, microarray probes for a particular gene are not always available, or they may cross-hybridize, and thus be omitted from coexpression calculations. In such cases, the second or third best platform would be selected based on the maxCOXSIM value. Thus we can expect higher maxCOXSIM values if an ideal reference platform can be used. Therefore, we normalized the maxCOXSIM values by the evolutionary distance. As a measure of coexpression data quality, we designed the reproducibility score, which is the median of the normalized maxCOXSIM for all of the guide genes in a data set. The reproducibility scores for the Arabidopsis coexpression data also showed improvement with each addition of new data, in good agreement with the GO scores (PCC = 0.88; Table 2).

Based on their potential as proxies for the species-specific GO score, we applied the codon score and the reproducibility score to assess all 14 coexpression data sets (Table 1). The codon score varied, with the highest scores for Zma-r (2.55) and Ath-m (2.29) and the lowest scores for Sly-m (1.07) and Mtr-m (1.08). Based on the codon score and the COXSIM values between the data sets (Fig. 1; Supplementary Fig. S1), we decided not to provide the Mtr-m coexpression data in the parallel view of gene coexpression, and thus they were not used in the calculation of reproducibility scores and were just made available as bulk-downloadable data. The reproducibility scores showed a similar trend to the codon score (PCC = 0.61), suggesting that both scores correctly assess the performance of each coexpression data set. Note that the association between gene expression and codon preference may vary among plant species, and thus the codon scores may not always be comparable among species.

### An example of lineage-specific coexpression supported by multiple platforms

Data from multiple platforms for a single species can confirm not only the existence, but also the absence, of coexpression. Among the five species with both microarray and RNAseq data, tomato had the lowest coexpression data (Sly-m, Sly-r) quality (Table 1; Fig. 1). Therefore, to examine monocot- and dicot-specific coexpression, we compared two monocots (rice and maize) and two dicots (Arabidopsis and soybean). Based on the reciprocal best hit method, there were 7,882 orthologous groups composed of one gene from each of the four species. Among the 7,882 orthologous groups, 1,548 had coexpression data from all eight data sets (microarray and RNAseq data for the four species). Before investigating the differences between the monocot and dicot species, we first compared the average coexpression strength in the four microarray data sets (Ath-m, Gma-m, Osa-m and Zma-m) and the four RNAseq data sets (Ath-r, Gma-r, Osa-r and Zma-r) by the geometric average of the mutual rank (MR) values (Supplementary Fig. S3A). The coexpression averages within each platform were similar (PCC = 0.70), and platform-specific bias was not observed. Because most gene pairs are not coexpressed, the peak of the distribution of the average coexpression is around MR = 10,000, which is approximately half of the number of genes in each data set. Next, we compared the average coexpression strength of dicots (Ath-m, Ath-r, Gma-m and Gma-r) and of monocots (Osa-m, Osa-r, Zma-m and Zma-r) (Supplementary Fig. S3B). The general trend of the average coexpression was similar to that seen in the comparison of platforms (Supplementary Fig. S3A). Most of the gene pairs in dicots and monocots were not coexpressed (approximate MR = 10,000). However, some of the coexpressed gene pairs showed strong coexpression in both monocots and dicots, indicating evolutionarily conserved coexpression. The distribution of the average coexpression was expanded relative to the platform-specific coexpression distribution (Supplementary Fig. S3A), suggesting the existence of monocot-specific and dicot-specific coexpression.

**Fig. 3:**
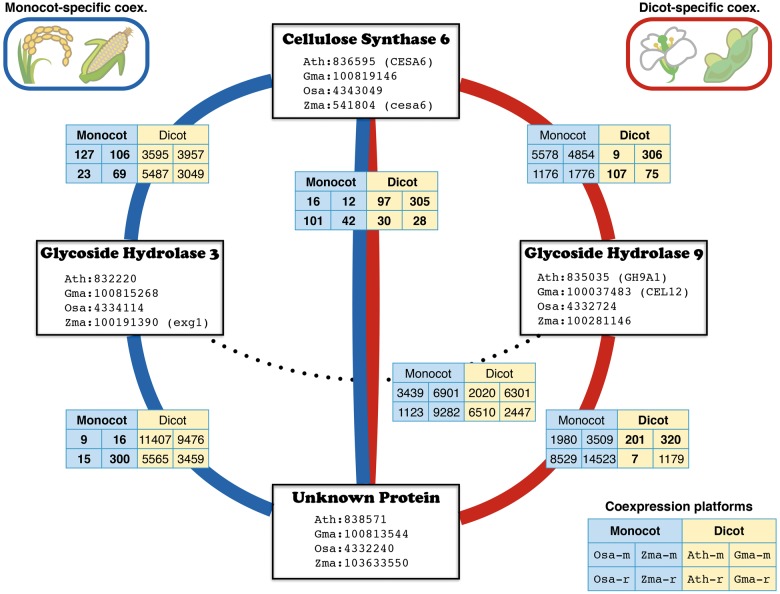
Example of lineage-specific coexpression. The genes encoding the four proteins cellulose synthase 6, glucoside hydrolase 3, glucoside hydrolase 9 and the protein of unknown function are represented as a coexpression network (also highlighted in Supplementary Fig. S3). Stronger coexpression (MR <500) is represented in bold. Blue and red edges represent monocot- and dicot-specific coexpression, respectively. All of the coexpression data for the eight data sets, with orthologous information, can be downloaded from ATTED-II (http://atted.jp/top_download.shtml).

Here, we focused on six gene pairs among four genes as examples of various coexpression patterns. Among the six gene pairs, one gene pair shows evolutionarily conserved coexpression, one gene pair is not coexpressed and the other four gene pairs show lineage-specific coexpression (Supplementary Fig. S3B). Interestingly, these examples revealed coexpression switching (Fig. 3). The gene encoding cellulose synthase 6 (CS6) was coexpressed with the gene for a protein of unknown function (Unk) in all eight data sets, and thus this unknown protein is very likely to be a cell wall rearrangement factor. In monocots, these two genes show coexpression with the gene for glycoside hydrolase 3 (GH3), which degrades the major hemicelluloses in monocots (xylan, arabinan and arabinoxylan; Minic 2008). On the other hand, in dicots, these two genes are coexpressed with the gene for glycoside hydrolase 9 (GH9), which degrades glucan and cellulose (Minic 2008). Monocots and dicots use distinct sets of genes to construct the different types of cell wall (Yokoyama and Nishitani 2004, Minic 2008). By using gene coexpression, the difference in the individual gene modules can be detected even in the common genes between the two lineages.

### Development of NetworkDrawer for subnetwork analyses

**Fig. 4:**
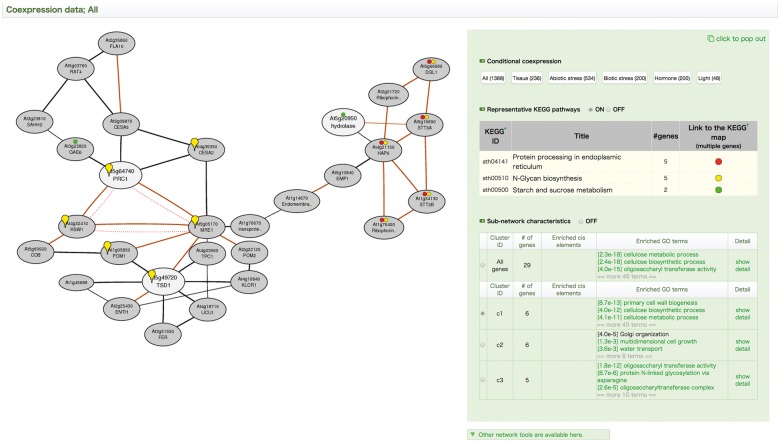
Update of NetworkDrawer with subnetwork analysis functions. NetworkDrawer output coexpression network for Arabidopsis (Ath-m) for a set of three query genes (white nodes; CS6/At5g64740, GH3/At5g20950 and GH9/At5g49720) with automatically retrieved coexpressed genes (gray nodes). Orange lines indicate highly reliable coexpression supported by data from the other data sets. Red dotted lines indicate protein–protein interaction. After construction of the coexpression network, subnetworks are automatically detected. For each subnetwork, enrichment tests are then conducted for GO annotations and heptamer *cis*-elements in the proximal promoter region [–300, –1] under Bonferroni correction. The subnetworks having at least one significantly enriched factor are shown on the right operation panel. Genes for a subnetwork of interest are highlighted by the yellow balloon marks, which can be manually selected with the right operating panel.

Network representation is a suitable method to provide an overview of the module structure of multiple gene relationships, such as coexpression. ATTED-II provides NetworkDrawer, a coexpression network drawing tool. Coexpression network is generally scale free (Jordan et al. 2004, van Noort et al. 2004), meaning that the user has little control over the density of the network. NetworkDrawer draws edges for the three genes with the strongest coexpression with every gene. This constraint provides a medium-density network for a variety of query gene sets. However, even with this drawing constraint, large gene networks easily become too complicated to investigate manually. Therefore, an automatic network analysis tool is needed. To find biologically meaningful subnetworks in large coexpressed gene networks, we implemented an automatic subnetwork detection and analysis workflow, as previously introduced in a mammalian coexpression database (Obayashi et al. 2013). After the coexpressed gene network is drawn, a subnetwork detection algorithm automatically initiates. All of the detected subnetworks are then checked for enrichment of GO biological process annotations and *cis*-elements. Fig. 4 shows an example NetworkDrawer output page for the CS6, GH3 and GH9 genes discussed above. The gene network based on the Ath-m data set reflects stronger coexpression between the CS6 (TSD1 in the Fig. 4) and GH3 (PRC1 in the Fig. 4) genes than between either of these genes and the GH9 gene. In this case, three biologically meaningful subnetworks were detected, which generally correspond to each query gene.

## Materials and Methods

### Construction of gene coexpression data

To calculate coexpression from the microarray-based data, we downloaded the GeneChip CEL files from ArrayExpress (Kolesnikov et al. 2015). Mapping from probe to gene was based on National Center for Biotechnology Information (NCBI) Gene Expression Omnibus platform files (Barrett et al. 2013). Probes only associated with a GenBank ID were re-mapped to the Entrez Gene ID using Blastn against the RefSeq sequences of the species (Altschul et al. 1997). Note that ATTED-II is now based on the NCBI Entrez Gene ID with the RefSeq sequences as the gene model for stable development for multiple species. The MR of the weighted PCC was used as the coexpression measure, as previously described (Obayashi and Kinoshita 2009). To calculate the coexpression in RNAseq-based data, we downloaded the Sequence Read Archive format data from the DNA Data Bank of Japan (Ogasawara et al. 2013), converted it to the FASTQ format and mapped it to the NCBI RefSeq sequences (Brown et al. 2015) using Bowtie (Langmead et al. 2009). Lower quality data were filtered out by the total mapped count<10,000,000. The mapped counts were summed for each gene model for use as the gene expression value. Genes with consistently low expression (highest count across all runs <100) were omitted. After conversion to a base-2 logarithm with a pseudo count of 0.125, quantile normalization was applied for each experiment, and the average expression levels were subtracted for each gene. Using all of the experimental data, PCCs between all gene pairs were calculated, and these values were then converted to MRs.

### Similarity of gene lists

We previously introduced the measure COXSIM to compare the coexpressed gene list from a guide gene *g* of interest $\left({\mathrm{list}}_{go}\right)$ and that from a reference guide gene *r* (*list*_*r*_0__) in a weighted manner (Obayashi et al. 2013). Because the gene compositions of ${\mathrm{list}}_{g_{0}}$ and ${\mathrm{list}}_{r_{0}}$ are different, we excluded the genes in both lists that lacked the corresponding genes in the other data set, resulting in *list_g_* and *list_r_*, respectively. The correspondence of genes between species was determined by the Blastp Reciprocal Best Hit method.

(1) $${\mathrm{COXSIM}}_{gr}=\overset{k}{\underset{i=1}{\sum }}n(i,{list}_{g},{list}_{r})/\overset{k}{\underset{i=1}{\sum }}i,$$

where *n*(*i*, *list_g_*, *list_r_*) is the number of genes in the top *i* genes in *list_g_* with corresponding genes in the top *i* genes in *list_r_*. We previously used 100 for *k*, meaning that we checked the gene correspondence of the top 100 coexpressed genes. However, the total number of genes in the gene list is different among the data sets, and thus the random inclusion rate of unrelated genes in the top 100 are different. Therefore, we have modified the number of genes in *k* to be the top 1% of all genes in *list_g_*. Typically, the *k* values are approximately 100 for different species comparisons and approximately 200 for same species comparisons.

Because the best reference guide gene is initially unknown, we checked all possible reference guide genes. The reference guide gene set *R* is composed of the Blastp best hit genes from one target species in every other species. The selection of the genes with the highest similarity is independent of the data set composition, and thus the best-hit gene is sometimes not available in the reference data set, and, in that case, we did not use the data set as the reference. When multiple data sets are available for the species including the guide gene *g*, the same gene in the other data set is also included in the reference guide gene set *R*. The COXSIM values are calculated between the target guide gene *g* and every reference gene *r* in *R*. The reference gene $\hat{r}$ that gives the maxCOXSIM value is regarded as the best reference guide gene.

(2) $${\mathrm{maxCOXSIM}}_{g}={\mathrm{COXSIM}}_{g\hat{r}}=\underset{r\in R}{\text{max}}\left[{\mathrm{COXSIM}}_{gr}\right]\;$$

To assess the statistical significance, the $maxCOXSIM_{g}$ value is then compared with the null distribution. To reflect the characteristics of the coexpression data, the actual $COXSIM_{gr}$ values between any combination of Arabidopsis gene *g* and rice gene *r* were used as the null distribution of the COXSIM values because almost all gene pairs are functionally independent (Supplementary Fig. S2). Based on this COXSIM distribution with Bonferroni correction for the 13 guide gene lists, the *p*-values of maxCOXSIM were determined. The thresholds of maxCOXSIM for *p* < 0.1, *p* < 0.01 and *p* < 0.001 were 0.081, 0.189 and 0.377, respectively.

### Reproducibility score based on similarity of coexpression data

As suggested from Fig. 2, the $maxCOXSIM_{g}$value is one representation of the quality of a guide gene, and thus the median $maxCOXSIM_{g}$value for every guide gene in a data set reflects the total quality of the data set. However, the $maxCOXSIM_{g}$value should be considered from the viewpoint of the adequateness of the selected reference guide gene $\hat{r}$. We designed the reproducibility score as a more accurate measure of data set quality:

(3) $$\mathrm{Reproducibility}\;=\underset{g\in platform}{\text{median}}\left[{\mathrm{COXSIM}}_{\text{g}\hat{r}}/{\mathrm{ReferenceAdequateness}}_{g\hat{r}}\right]$$

Because different data sets from the same species are the best reference to check technical reproducibility (Supplementary Figs. S1, S2), the adequateness of the reference guide gene in the same species should be the highest, whereas that of the reference guide gene in the evolutionarily most distant species should be the lowest. We hypothesized that the conservation of gene coexpression could be approximated by using the conservation of the guide gene sequences. Based on this idea, we used the conservation ratio between protein sequences of the target guide gene *g* and the selected reference guide gene $\hat{r}$ to measure the adequateness of the selected guide gene $\hat{r}$ as the reference.

(4) $$ReferenceAdequateness_{g\hat{r}}=\frac{\left(Blastp\;bitscore\;from\;g\;to\;\hat{r}\right)}{\left(Blastp\;bitscore\;from\;g\;to\;g\right)}$$

The reproducibilityscore, which is the median of the normalized maxCOXSIM for all of the guide genes in a data set in Equation 3, is used as the coexpression quality value of the data set (Tables 1, 2).

### Predictive performance of GO terms by gene coexpression data

Using the GO biological process annotations downloaded from NCBI (August 10, 2015, gene2go), the Arabidopsis coexpression data were assessed (Table 2). Owing to the differences in the relevance of GO terms to our purposes along with their hierarchical topology, we selected particular GO terms for coexpression evaluation, as described previously (Kinoshita and Obayashi 2009). We selected GO terms associated with 5–20 genes with comparable information content, resulting in 1,197 GO biological process terms for Arabidopsis. The genes associated with at least one selected GO term were then used in this assessment, resulting in 3,708 and 4,163 genes for the Ath-m and Ath-r data sets, respectively. All of the genes within each data set were divided into two groups, those sharing at least one GO term with another gene. The differences in the distribution of the degrees of coexpression were assessed using the partial receiver operating characteristic (ROC) area under the curve (AUC)_0.01_ (McClish 1989). Note that this GO score scheme is not applicable for most of the other species. For example, only 27 GO biological process terms have been identified for rice.

### Coincidence score with codon similarity

Protein-coding sequences were retrieved from NCBI RefSeq (Brown et al. 2015). For each gene, the 61-dimensional vector was constructed from the number of codons in the protein-coding sequence. When multiple RefSeq sequences were available for a gene, the longest sequence was used for the calculation of codon usage. The PCCs between the vectors of any two genes were calculated and then converted to MRs, which were used as the codon usage similarity index. Codon similarity tables are also downloadable from the ATTED-II bulk-download page (http://atted.jp/ top_download.shtml) in the same format as the coexpression tables.

## Supplementary data

Supplementary data are available at PCP online.

## Funding

This research was supported by the Japan Science and Technology Agency (CREST research project grant No. 11102558 to T.O.); Grants-in-Aid for Innovative Areas (grant No. 24114005 to T.O.), Scientific Research (grant Nos. 15K20863 to Y.A. and 15K18464 to T.O.) and Publication of Scientific Research Results (grant No. 15HP8044 to T.O.).

## Supplementary Material

Supplementary Data

## Abbreviations

AUC: area under the curve
CS6: cellulose synthase 6
GH3: glycoside hydrolase 3
GH9: glycoside hydrolase 9
GO: Gene Ontology
MR: mutual rank
PCC: Pearson’s correlation coefficient
RNAseq: RNA sequencing
