# Supplementary material for: ATTED-II in 2016: A Plant Coexpression Database Towards Lineage-Specific Coexpression
Source: Plant Cell Physiol. 2015 Nov 6;57(1):e5. doi: 10.1093/pcp/pcv165 (PMC4722172; doi:10.1093/pcp/pcv165)
Supplement: Supplementary Data [file supp_57_1_e5__index.html]

ATTED-II in 2016: A Plant Coexpression Database Towards Lineage-Specific Coexpression — ATTED-II in 2016: A Plant Coexpression Database Towards Lineage-Specific Coexpression — Supplementary Data 

# ATTED-II in 2016: A Plant Coexpression Database Towards Lineage-Specific Coexpression

## Supplementary Data

files

- Supplementary Data - pdf file
- Supplementary Data - pdf file
- Supplementary Data - pdf file
