# Supplementary material for: ATTED-II in 2016: A Plant Coexpression Database Towards Lineage-Specific Coexpression
Source: Plant Cell Physiol. 2015 Nov 6;57(1):e5. doi: 10.1093/pcp/pcv165 (PMC4722172; doi:10.1093/pcp/pcv165)
Supplement: Supplementary Data [file supp_pcv165_pcp-2015-e-00489-File006.pdf]

		Reference Data													
		Zma-m	Zma-r	Osa-m	Osa-r	Mtr-m	Sly-m	Sly-r	Bra-r	Ath-m	Ath-r	Vvi-m	Ppo-m	Gma-m	Gma-r
Target Data	Zma-m	-	0.161	0.051	0.034	0.000	0.000	0.017	0.014	0.026	0.017	0.015	0.009	0.013	0.020
	Zma-r	0.161	-	0.056	0.045	0.003	0.019	0.022	0.021	0.034	0.023	0.022	0.021	0.027	0.027
	Osa-m	0.052	0.055	-	0.146	0.000	0.019	0.019	0.019	0.032	0.022	0.022	0.022	0.028	0.023
	Osa-r	0.034	0.043	0.146	-	0.000	0.011	0.016	0.016	0.024	0.018	0.019	0.016	0.022	0.020
	Mtr-m	0.000	0.000	0.000	0.000	-	0.000	0.004	0.002	0.012	0.006	0.000	0.000	0.000	0.018
	Sly-m	0.000	0.017	0.017	0.011	0.000	-	0.083	0.013	0.030	0.020	0.017	0.012	0.010	0.022
	Sly-r	0.018	0.024	0.020	0.018	0.008	0.083	-	0.019	0.029	0.023	0.022	0.021	0.025	0.024
	Bra-r	0.017	0.024	0.020	0.019	0.004	0.018	0.020	-	0.062	0.040	0.019	0.020	0.022	0.023
	Ath-m	0.030	0.040	0.036	0.029	0.017	0.035	0.030	0.059	-	0.226	0.031	0.039	0.042	0.038
	Ath-r	0.022	0.027	0.025	0.022	0.011	0.027	0.024	0.040	0.226	-	0.023	0.026	0.028	0.027
	Vvi-m	0.015	0.023	0.022	0.019	0.000	0.020	0.020	0.017	0.028	0.020	-	0.023	0.026	0.023
	Ppo-m	0.014	0.025	0.025	0.020	0.000	0.020	0.023	0.022	0.042	0.027	0.027	-	0.032	0.028
	Gma-m	0.013	0.026	0.026	0.022	0.000	0.010	0.022	0.020	0.035	0.023	0.023	0.024	-	0.121
	Gma-r	0.022	0.030	0.025	0.022	0.022	0.026	0.024	0.022	0.037	0.025	0.025	0.026	0.121	-

Supplementary Fig. S1: Median *COXSIM* values for target to reference data sets.
Because *COXSIM* values are not exactly symmetric, the median of *COXSIM* for a data set pair is not exactly symmetric. The average values of the median *COXSIM* from one target to one reference and vise versa were used for the dendrogram in Figure 1. Similar coexpression data sets are highlited by color.
