# Supplementary material for: ATTED-II in 2016: A Plant Coexpression Database Towards Lineage-Specific Coexpression
Source: Plant Cell Physiol. 2015 Nov 6;57(1):e5. doi: 10.1093/pcp/pcv165 (PMC4722172; doi:10.1093/pcp/pcv165)
Supplement: Supplementary Data [file supp_pcv165_pcp-2015-e-00489-File007.pdf]

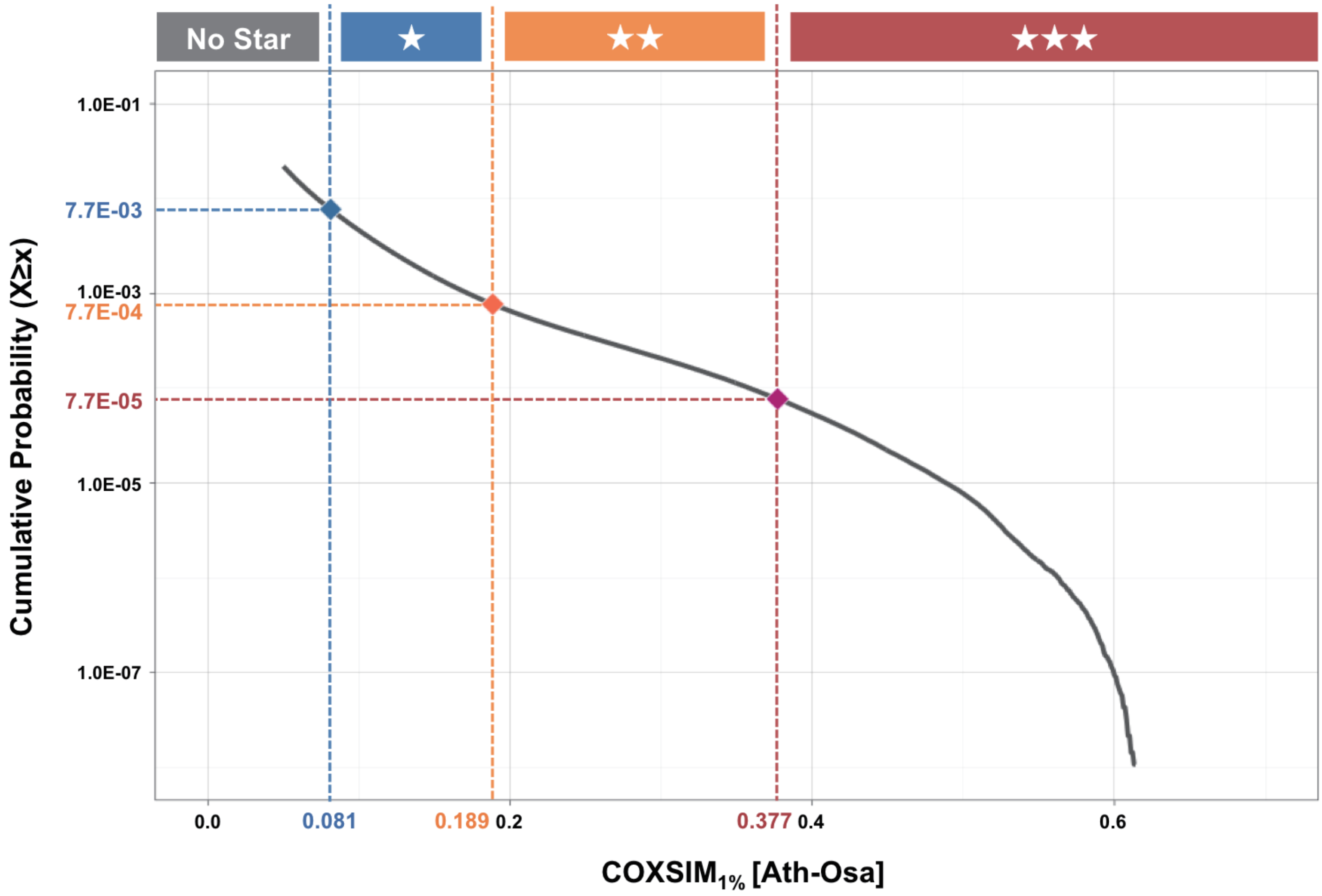

Supplementary Fig. S2: The null distribution of *maxCOXSIM* value based on actual *COXSIM* values. All combination between Arabidopsis and rice genes were used for the null distribution of *COXSIM* values. 7.7E-03, 7.7E-04 and 7.7E-05 are corresponding to p=0.1, p=0.01 and p=0.001, respectively, under Bonferroni correction for the 13 reference platforms. The supportability levels are represented as stars, where no star is the lowest and three stars is the highest.
