# Supplementary material for: ATTED-II in 2016: A Plant Coexpression Database Towards Lineage-Specific Coexpression
Source: Plant Cell Physiol. 2015 Nov 6;57(1):e5. doi: 10.1093/pcp/pcv165 (PMC4722172; doi:10.1093/pcp/pcv165)
Supplement: Supplementary Data [file supp_pcv165_pcp-2015-e-00489-File008.pdf]

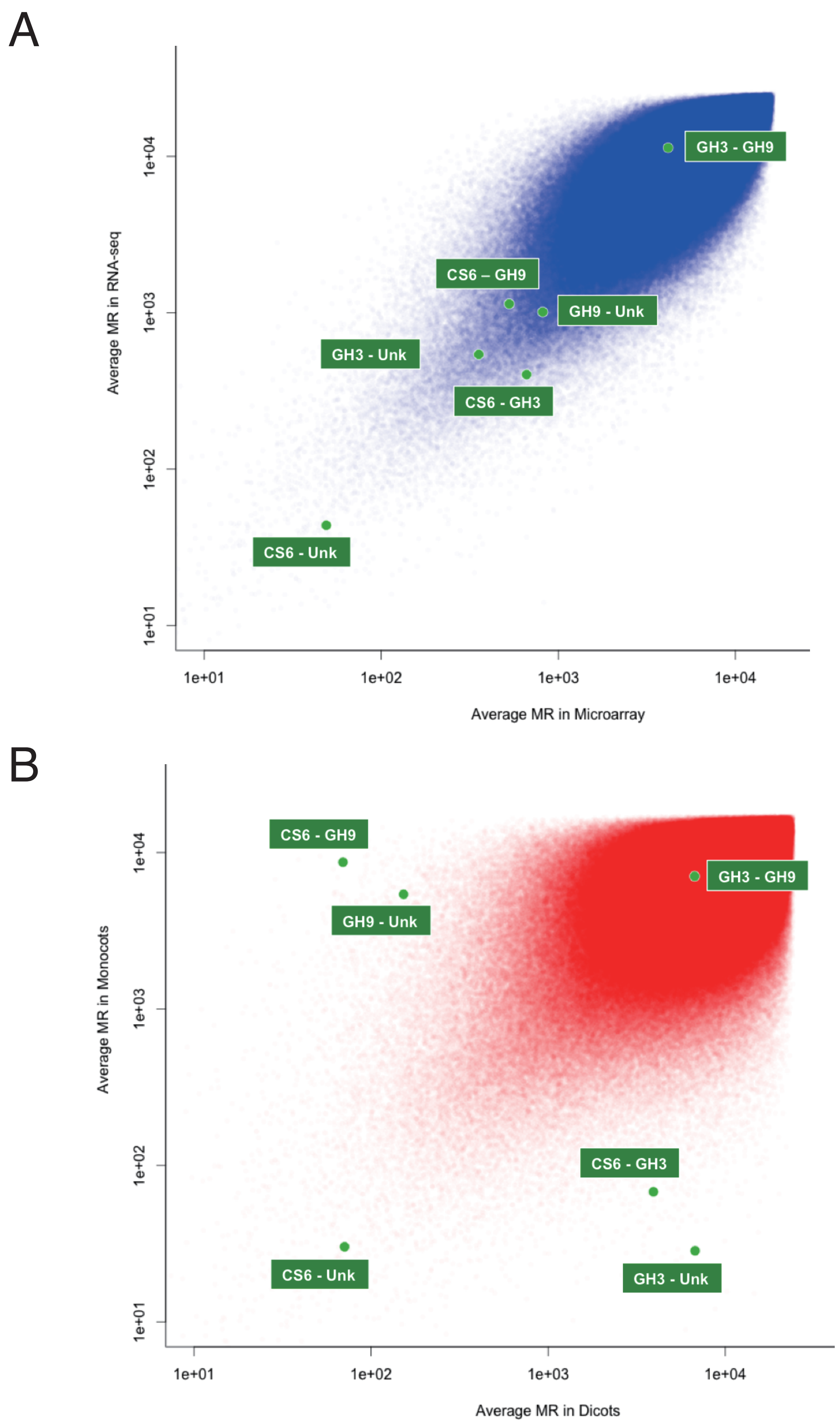

Supplementary Fig. S3: Existence of lineage-specific coexpression. (A) Comparison between the geometric averages of the coexpression values for the microarray (Ath-m, Gma-m, Osa-m, Zma-m) and RNAseq (Ath-r, Gma-r, Osa-r, Zma-r) platforms showing no strong differences in coexpression between the two platforms. (B) The geometric averages of coexpression values for dicot (Ath-m, Ath-r, Gma-m, Gma-r) and monocot (Osa-m, Osa-r, Zma-m, Zma-r) species showed more diverse distribution, reflecting lineage-specific coexpression.
